# Inhaled Antibiotic and Biologic Formulations Targeting *Pseudomonas aeruginosa*

**DOI:** 10.3390/pharmaceutics18020162

**Published:** 2026-01-26

**Authors:** Prodip Kumar Baral, Jack Dummer, Daniel Pletzer, Shyamal C. Das

**Affiliations:** 1 School of Pharmacy, Faculty of Health Professional Programmes, University of Otago, Dunedin 9054, New Zealand; barpr688@student.otago.ac.nz; 2 Department of Microbiology and Immunology, Faculty of Biomedical Sciences, University of Otago, Dunedin 9054, New Zealand; daniel.pletzer@otago.ac.nz; 3 Department of Pharmacy, Noakhali Science and Technology University, Noakhali 3814, Bangladesh; 4 Department of Medicine, Faculty of Medicine, University of Otago, Dunedin 9054, New Zealand; jack.dummer@otago.ac.nz

**Keywords:** pulmonary delivery, nanoparticles, microparticles, nano-in-micro particles, dry powder inhaler, antimicrobial peptides, bacteriophage

## Abstract

Lower respiratory tract infections caused by *Pseudomonas aeruginosa* are a global concern. Patients with chronic lung diseases such as cystic fibrosis and non-cystic fibrosis bronchiectasis often do not receive adequate antibiotic delivery through conventional routes. *P. aeruginosa* employs several mechanisms, including biofilm formation and efflux pumps to limit the accumulation of bactericidal drug concentrations. Direct drug delivery to the lung epithelial lining fluid can increase antibiotic concentration and reduce treatment failure rates. This review discusses current research and developments in inhaled antibiotic formulations for treating *P. aeruginosa* infections. Recent studies on particle engineering for the dry powder inhalers of antibiotics emphasized three fundamental principles of development: micro, nano, and nano-in-microparticles. Carrier-free microparticles showed potential for high-dose delivery but suffered from poor aerosolization, which could be improved through a drug–drug combination. Amino acids in a co-spray-dried system improved powders’ aerodynamics and reduced moisture sensitivity while incorporating the chitosan/poly(lactic-co-glycolic acid) (PLGA)-modified release of the drug. Nano-in-microsystems, embedding lipid carriers, showed improved antibiofilm activity and controlled release. We also highlight emerging biologics, including antibacterial proteins/peptides, vaccines, bacteriophages, and probiotics. Research on antibiotics and biologics for inhalation suggests excellent safety profiles and encouraging efficacy for some formulations, including antimicrobial peptides and bacteriophage formulations. Further research on novel molecules and synergistic biologic combinations, supported by comprehensive animal lung safety investigations, will be required in future developments.

## 1. Introduction

Lung infections are one of the leading causes of mortality [1], and *Pseudomonas aeruginosa* is responsible for a significant healthcare burden, with a 7% prevalence among all healthcare-associated infections [2]. Its metabolic adaptability facilitates its role in hospital-acquired and immunocompromised infections [3]. This bacterium is also an opportunistic colonizer in long-term conditions such as cystic fibrosis and non-cystic fibrosis bronchiectasis. *P. aeruginosa* has obtained the attention of the World Health Organization because of the frequency of biofilm formation and resistance development due to exposure to antibiotics. It is designated as a “High priority” bacterium [4].

Direct inhalation of antibiotics for *P. aeruginosa* respiratory tract infections can offer several advantages, including high-dose deposition at the site of infection, reduced systemic toxicity, and controlled drug release. Formulation scientists aim to enhance antibiotic delivery via particle engineering techniques to maintain optimal size, size distribution, shape, texture, charge, hygroscopicity, and rheology to increase deposition in the bronchioles and alveoli [5].

The growing resistance of *P. aeruginosa* to antibiotics used in pulmonary infections highlights the urgent need to explore novel treatment options beyond conventional broad-spectrum antibiotics. To overcome drug resistance, current drug formulation developments include antibacterial biologics, such as antibacterial proteins/peptides, bacteriophages, probiotics, and antibodies [6,7,8,9]. Additionally, novel nanotechnology-based formulation techniques have been used to evade bacterial resistance and improve drug uptake into bacterial cells [10]. Furthermore, research has shown synergy between biologics and antibiotics: several peptides and peptidomimetics, such as KR-12-a5, TM18, and DJK-5, have been explored as adjuvants that might have antibiofilm activity and could act synergistically with conventional antibiotics [11,12,13,14].

A variety of inhaled formulations with activity against *P. aeruginosa* have been developed using a range of techniques. A comprehensive understanding is required to refine those techniques and to further develop this promising area of work. This review discusses recent advances in inhalable formulations of antibiotics and biologics for the treatment of bacterial lung diseases caused by *P. aeruginosa*.

## 2. *P. aeruginosa* Lung Diseases

*P. aeruginosa* can cause hospital-acquired pneumonia, can be responsible for infective exacerbations of cystic fibrosis (CF), non-CF bronchiectasis, and COPD, and it can cause pneumonia in immunosuppressed patients. Current treatment strategies are suboptimal, requiring prolonged courses of systemic (often intravenous) antibiotics. The antibiotics used for treatment include β-lactams (piperacillin–tazobactam, ceftazidime, and cefepime), carbapenems (imipenem, meropenem), aminoglycosides (amikacin, tobramycin), and fluoroquinolones (ciprofloxacin, levofloxacin) [15,16]. However, they often fail to achieve bactericidal concentrations at the infection site in the lungs [17,18]. In CF and bronchiectasis, eradication rates remain suboptimal, with frequent recurrence and progressive lung damage. COPD patients experience exacerbations linked to *P. aeruginosa* colonization. In HAP/VAP, MDR strains complicate therapy, prolong hospital stays, and increase mortality. These challenges highlight the unmet need for the targeted lung delivery of antibiotics and biologics.

### 2.1. Hospital-Acquired Pneumonia

*P. aeruginosa* is associated with various acute infections, often in healthcare settings, including nosocomial pneumonia, ventilator-associated pneumonia, and immunodeficiency-associated pneumonia [19]. The organism causes nosocomial infection that substantially increases mortality rates, hospital stays, and treatment costs [20]. The risk of hospital-associated infections increases by 6–20 times when critically ill patients are placed on ventilators, and *P. aeruginosa* is a major causative pathogen contributing to high mortality rates [21,22]. As an opportunistic pathogen, it also causes lung infections in immunosuppressed individuals, including those who have undergone organ transplantation, burn treatment, or cancer therapy, and people with HIV infection [23].

### 2.2. Infection in Cystic Fibrosis (CF)

CF is an inherited disorder characterized by the impaired clearance of thick, dehydrated mucus from the respiratory tract. This static mucus facilitates bacterial growth. People with CF develop bronchiectasis: widened, damaged airways associated with an increased volume of respiratory secretions and recurrent respiratory tract infections. *P. aeruginosa* is the most prevalent respiratory pathogen in adults with cystic fibrosis (prevalence > 60%), and it is responsible for an accelerated decline in lung function, worse radiological appearances, worse nutrition, and faster progression to end-stage disease [24]. Eradication of the organism is challenging, and the failure rate of current eradication therapy (usually with combined intravenous and nebulised antibiotic therapy) is 10–40% [25]. Several inhaled drug formulations have been approved for the treatment of *P. aeruginosa* infections in CF patients. The first FDA-approved antibiotic for this condition was tobramycin inhalation solution (TOBI^®^), introduced in 1975. In 2013, the only dry powder inhaler of tobramycin (TOBI^®^ Podhaler™) was approved, followed by the approval of an inhalable aztreonam solution in 2014 [26].

### 2.3. Infection in Non-CF Bronchiectasis

Non-CF bronchiectasis has multiple causes, including post-infective, COPD, asthma, and tuberculosis, but most commonly, the cause is idiopathic or unknown. *P. aeruginosa* is one of the most common organisms present in people with non-CF bronchiectasis and is present in approximately 25% of patients. Its presence is associated with worse clinical outcomes, including frequency of respiratory tract infections, hospitalisations, decline in lung function, and mortality [27]. Nebulised colistin or gentamicin can be considered in order to reduce the frequency of respiratory tract infection in these patients [28].

### 2.4. Infection in Chronic Obstructive Pulmonary Disease (COPD)

COPD is a leading cause of respiratory failure, being responsible for more than 3 million deaths annually worldwide [29]. COPD is caused by chronic exposure to pollutants, irritants, and smoke, which damage the respiratory system, leading to persistent inflammation, airway obstruction, and a gradual decline in quality of life [30,31]. Epidemiological investigations have documented *P. aeruginosa* colonization in 10–35% of COPD cases, highlighting its clinical significance in disease progression [32]. The bacterium is responsible for 5–10% of COPD exacerbations, contributing to increased mortality in affected individuals [33,34].

## 3. Characteristics of *P. aeruginosa* Associated with Pulmonary Infections

**Figure 1:**
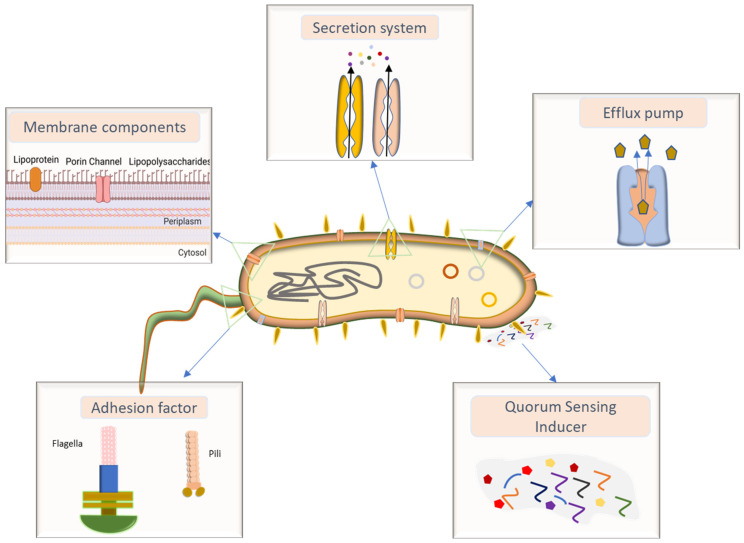
The types of structural and functional components of *P. aeruginosa* responsible for lung infections: adhesion factor, membrane components, secretion system, efflux pump, and quorum-sensing inducer.

*P. aeruginosa* is a Gram-negative, rod-shaped bacterium with a range of factors enabling it to infect the respiratory tract and to develop resistance to antibiotics (Figure 1). It adheres to the airway epithelium using flagella, pili, and membrane-associated constituents such as lipopolysaccharides and adhesion factors [35], and it can form biofilms in the airways [36].

*P. aeruginosa* has several virulence factors that are associated with severe pulmonary infections. Bacterial outer membrane porins restrict the permeability of antibiotics, while lipopolysaccharide (LPS) modifications confer resistance to drugs [37]. Efflux pumps actively expel the drugs, reducing intracellular drug concentrations and promoting multidrug resistance [38]. Secretion systems release toxins that damage airway epithelium, exacerbate inflammation, and accelerate disease progression. Additionally, the formation of biofilm creates a physical and metabolic barrier to drug penetration. These mechanisms are described in greater detail below. Together, they act to reduce the effectiveness of systemic antibiotic therapy to the point that treatment failure is common. Much higher local antibiotic concentrations are needed and could be achieved by direct delivery of inhalable formulations engineered for bronchioalveolar deposition, enhanced mucus and biofilm penetration, and controlled drug release.

### 3.1. Adhesion Factors

*P. aeruginosa* possesses a single, polar flagellum responsible for motility and attachment to the epithelial lining and powered by two torque-generating flagellar stators [39]. The flagellin protein can induce the generation of proinflammatory mediators by airway epithelial cells [40]. The pili are associated with twitching motility on solid surfaces and genetic material transfer [41]. The flagellar cap protein, FliD, and type IV pili (TFP) bind to glycosphingolipids located on the respiratory epithelium and to heparan sulfate proteoglycans and N-glycans of the basolateral surface [35,42]. Immature and injured epithelial cells express a high level of surface molecules, and interaction with FliD and type IV pili induces the transcription and mobilization of asialoGM1 and Toll-like receptors (TLR-2 and TLR-5) [42]. Pili serve as receptors for bacteriophage entry, facilitating viral transfer into bacterial cells and subsequent lysis [43]. However, type IV pilus glycosylation reduces the uptake of the phage virus, acting as a bacterial defence mechanism [44].

### 3.2. Membrane Components

The outer membrane of Gram-negative bacteria exhibits some permeability due to the presence of certain lipoproteins and porin channels. These channels are responsible for the passive and active transport of small molecules, cellular adhesion, and activation of transmembrane receptors [45]. The outer membrane porin F (OprF) permits the nonspecific diffusion of extracellular molecules and xenobiotics, but the OprF of *P. aeruginosa* exhibits very low permeability, substantially reducing the permeability of hydrophobic drugs [46]. Two other porins (OprB and OprB2) are associated with glucose diffusion, phosphate/pyrophosphate uptake by two small membrane proteins (OprG and OprH), and two porins (OprP and OprO) [45]. The overexpression of *oprH* causes resistance to cationic peptides and some antibiotics, including aminoglycosides and polymyxin B [47]. Similarly, OprJ, OprM, and OprN contribute to bacterial multidrug resistance [48]. However, OprI is a potential receptor that internalizes cationic antimicrobial proteins/peptides such as LL-37 and defensins [49].

The outer membranes consist of lipopolysaccharide (LPS), which comprises the lipid A moiety, core oligosaccharides, and the O antigen. These components are involved in the immune response and interaction with antibodies. *P. aeruginosa* strains cultured from the airways of people with CF synthesize palmitate and aminoarabinose, containing a unique hexa-acylated lipid A, and are recognized by the TLR-4 complex of host cells and propagated immune reaction [50]. Nevertheless, the aminoarabinosylation of lipid A was found to be resistant to the cell membrane inhibitor drug colistin [51]. Cigana et al. revealed the genetic (*pagL*) mutation responsible for lipid A modification, helping it to evade immune detection [52]. The O-specific antigen also acts as a receptor for bacteriophages (e.g., *P. aeruginosa* phage K8). A thin layer of peptidoglycan is located just beneath the membrane. The muropeptides of peptidoglycan trigger nucleotide-binding oligomerization domain (NOD)-2 of the intracellular innate immune receptor [53].

### 3.3. Secretion Systems

*P. aeruginosa* secretes virulence factors into the extracellular space and targeted cells via five types of secretion systems. The major factors related to *P. aeruginosa* infection are secreted through either the type II (T2SS) or type III (T3SS) secretion systems. T2SS releases exotoxin A, elastase, protease, and phospholipase C using a two-step process from the cytosol to the periplasmic space and then to the extracellular space, contributing to lung injury and disease severity [54]. T3SS injects toxic proteins (ExoS, ExoT, ExoU, and ExoY) into eukaryotic host cells and contributes to chronic pulmonary infections and worse clinical consequences [55].

### 3.4. Efflux Pump

The efflux pump is considered a crucial transmembrane complex protein responsible for drug resistance, particularly in Gram-negative bacteria. *P. aeruginosa* exhibits drug resistance through four basic efflux systems: MexCD-OprJ, MexAB-OprM, MexEF-OprN, and MexXY [56]. These pumps expel xenobiotics, including antibiotics, from the bacterium. Exposure to xenobiotics induces mutations in the genes of the pump proteins and produces multidrug-resistant *P. aeruginosa* with an altered virulence, stress response, and biofilm formation [57].

### 3.5. Quorum Sensing (QS) and Biofilm Formation

The cell-to-cell communication that regulates gene expression and contributes to bacterial resistance development is called quorum sensing. The bacteria can form colonies using bacterial extracellular polymeric substances (EPSs), such as alginate, proteins, and extracellular DNA, which facilitate the transfer of sensing molecules [58]. The creation of biofilms by colonies in the lungs is a significant virulence factor, which can contribute to chronic pulmonary infection; many antibiotics show poor penetration of *P. aeruginosa* biofilms, and bacteria deep within the biofilm may be dormant and tolerant to most antibiotics [59]. Moreover, the quorum-sensing inducer expresses more efflux pumps [60].

Bacteria have three ways of QS, Las, Rhl, and Pqs, and can generate three polar autoinducers, including 3-oxo-C12-homoserine lactone (3-oxo-C12-HSL), *N*-butyryl homoserine lactone (C4-HSL), and 2-heptyl-3-hydroxy-4-quinolone, respectively [61]. The pathways are interconnected to produce several factors responsible for infection progression, such as siderophores, pyocyanin, elastase, and proteases [62].

## 4. Formulations of Pulmonary Antibiotics

### 4.1. Nebulized Antibiotics

In the early stages of antibiotic inhalation therapy, injectable solutions were repurposed for nebulization. Usually, the nebulizer solutions were not extensively modified beyond enhancing drug solubility, often requiring solubility-enhancing co-solvents or solubilizing agents [63]. A successful inhaled antibiotic delivery requires more than deep lung deposition; the formulation must also ensure adequate antimicrobial activity and the effective penetration of bacterial biofilms.

Advancements in nebulized antibiotics include the development of nanoformulations using nanoparticles as drug carriers. Recently, a nanoformulation of dextran single-chain nanoparticles (KuDa) and tobramycin (KuDa-tob) was developed. The KuDa nanoparticles neutralized the positive charge of the drug and facilitated drug diffusion through the mucus and biofilm. Nebulization of KuDa-tob reconstituted in the ideal range size for lung deposition, with a mass median aerodynamic diameter (MMAD) of 2.2 μm [64].

Ciprofloxacin-loaded PLGA nanoparticles have also been developed for pulmonary delivery and enhanced biofilm penetrability to treat *P. aeruginosa* infections [65]. This method produced particles smaller than 1 µm, where the encapsulation efficiency remained above 65%; however, antibiofilm activities were not evaluated.

Lipid-based nano-carriers have also been developed for nebulized antibiotics. Azithromycin was loaded into archaeolipids (NanoARC) that exhibited antibiofilm effects. Notably, a 2 mL dose of NanoARC-AZ (15 μg/mL AZ) delivered over five minutes was sufficient to achieve and maintain effective lung concentrations [66]. Another study developed two types of ciprofloxacin-loaded nanoliposomes based on size: NLC-S and NLC-L. In terms of aerosolization, no substantial difference was observed between them, but NLC-S significantly slowed the release of ciprofloxacin [67]. In another study, nanostructured lipid carriers were used to entrap levofloxacin, combining a mucolytic enzyme, DNase type I, and exhibited controlled release (60% in 4 h) [68]. Although these lipid-based nanoformulations did not achieve a high amount of drug encapsulation, they are important in demonstrating that nanoformulations can achieve efficient delivery to the deep airways and can enable controlled and sustained drug release.

Despite the widespread use of nebulized drugs, it is important to note that they suffer from significant limitations. Administration times are long, devices are bulky and not easily portable for travel, and set-up and cleaning can be a challenge. They can be an imposition on the daily routine of patients, who can find adherence to therapy challenging and would prefer to use inhalers if these were available [69]. Furthermore, aqueous formulations of some antibiotics are unstable, limiting options for administration and storage, and nosocomial infection can occur via the equipment [70,71].

### 4.2. Dry Powder Inhalers (DPIs)

**Figure 2:**
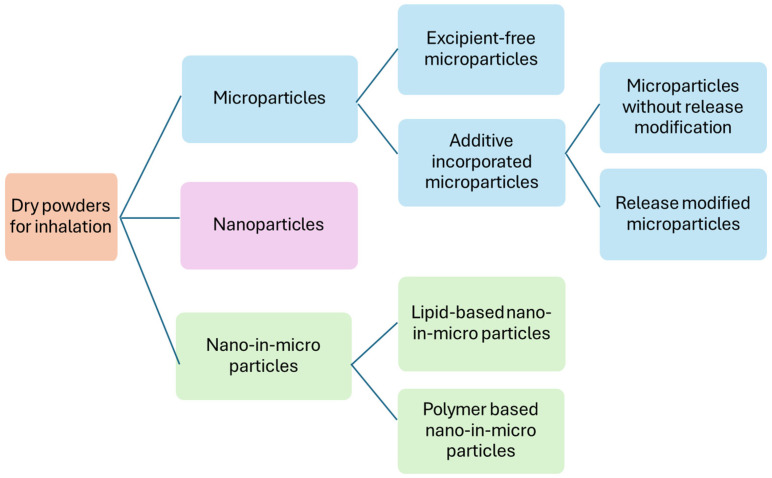
Different types of dry powder formulations developed for pulmonary delivery against *P. aeruginosa*.

A wide range of antibiotics has been explored for DPI formulation, including cephalosporins, monobactams, carbapenems, aminoglycosides, macrolides, and fluoroquinolones. Ciprofloxacin and levofloxacin have received the most research attention for anti-*P. aeruginosa* activity. The DPI formulation strategies employed in the literature can be broadly categorized into three main approaches (Figure 2): (1) microparticles, (2) nanoparticles, and (3) nano-in-microparticles.

#### 4.2.1. Microparticles

In general, microparticles range in geometric size from 1 to 1000 µm. Instead of geometric diameter (d_g_), the aerodynamic diameter (d_aer_) is appropriate for defining aerosolization, and particles in the d_aer_ of 1–5 µm are considered suitable for lower respiratory delivery deposition [72].

##### Excipient-Free Microparticles

This technique is ideal for high-dose drug delivery, particularly for antibiotics, since eliminating carrier materials increases the proportion of active pharmaceutical ingredients in DPI powders. Spray drying has been widely tested to produce excipient-free antibiotic particles (Table 1). Optimizing the spray drying process parameters remained a strategy to improve aerosolization without using additives [73,74]. In addition, co-spray drying antibiotics in combination therapy improved aerosolization [75,76,77,78]. The use of colistin with tobramycin improved the emitted dose and fine particle fractions of tobramycin at 1:1 and 1:5 ratios [77]. Similarly, the production of a co-amorphous system combining the antioxidant quercetin with ciprofloxacin improved fine particle fractions. However, regarding the fine particle doses of the monotherapy, the combination may not show significant differences [76].

Spray freeze drying (SFD) has been used to produce porous microparticles with very low aerodynamic diameters. Cheng et al. produced excipient-free tobramycin SFD powder with mass median aerodynamic diameters of 1.30 µm. Their product exhibited very high FPF (83.31%), although the study observed a limitation of their experimental equipment in optimizing the preparation using an increased feeding rate [79]. Electrospray, a novel method, was used to produce microparticles of azithromycin with a particle size around 2.5 µm, but the fine particle fraction remained below 20% [80]. These recent techniques avoid heat application, making them suitable for processing thermolabile drugs.

**Table 1:** Studies of excipient-free microparticles of antibiotics for dry powder inhaler (DPI) targeting *P. aeruginosa* infection from 2015 to 2025.

Drug Name	Preparation Methods	Highlights of Findings	Ref.
Aztreonam	SD	Excellent flowability and aerodynamic property; particle size < 4.46 μm; FPF 45%	[81]
Amikacin	SD	PDI was not considerably improved; ethanol improved the morphology of the particles	[82]
Azithromycin	Electrospray	High yield value; particle size around 2.5 µm; retained bactericidal effect and biocompatible	[80]
Ciprofloxacin	SD	Spherical, crystalline, dimpled particles; good yield with free flowability	[74]
Tobramycin	SFD	MMAD of optimized formulation was 1.30; FPF 83.31%; safety in vivo study; enhanced efficacy in the infected mouse compared to IV route	[79]
Meropenem-colistin	SD	Meropenem-colistin had synergistic antibacterial effects against clinical strains of *P. aeruginosa*; the co-formulation improved the aerosolization properties	[75]
Tobramycin—colistin	SD	Improved aerosolization performance; enhanced stability; prevented moisture-induced particle agglomeration	[77]
Clarithromycin—*N*-acetylcysteine	SD	Suitable MMAD of particles; solubility of the drug was improved	[78]
Ciprofloxacin—quercetin	SD	Enhanced aerodynamic deposition (72–94%) and FPF (48–65%); improved stability	[76]

FPF, fine particle fraction; MMAD, mass median aerodynamic diameter; PDI, polydispersibility index; SD, spray drying; SFD, spray freeze drying.

##### Additive Incorporated Microparticles

*a.*  *Microparticles without release modification*

Amino acids and mono-/di-saccharides were primarily incorporated as additives to tailor particle properties for specific physicochemical characteristics (Table 2). Amino acids (valine, leucine, methionine, phenylalanine, tryptophan, and arginine) significantly contributed to both enhancing aerosolization and reducing moisture content in the powder [83,84,85]. Micronized lactose and mannitol were used to improve the flow of their powders by physical mixing [86,87]. Several studies revealed that leucine played a crucial role in improving particles’ aerodynamics by reducing cohesiveness in co-spray dried powder [83,84,85]. In addition, optimization of the pH of the leucine-containing feed improved the particles’ aerodynamic parameters [88]. The study found the best fine particle fraction of co-spray dried levofloxacin at pH 5.98.

Tryptophan is the most hydrophobic of the essential amino acids [89] and has been used for drug-stabilizing using an equal proportion of ceftazidime under <15% humidity and 25 °C [83]. Chang et al. demonstrated the moisture-protection efficacy of D-amino acids (D-Methionine and D-Tryptophan) due to their recrystallization in the presence of moisture. Additionally, D-amino acids showed significant improvement of antibiofilm activity when combined with ciprofloxacin compared with ciprofloxacin alone [90]. Current particle-engineering research is increasingly focused on co-spray-dried antibiotic microparticles incorporating amino acids as functional additives to enhance aerosolization and stability without altering the drug release profile. This technique might be commercially feasible and is not expected to adversely affect the lung safety of the drugs.

**Table 2:** Studies of microparticles of antibiotics with additives (amino acids, mono-/di-saccharides) for dry powder inhaler (DPI) targeting *P. aeruginosa* infection from 2015 to 2025.

Drug Name	Preparation Method	Additives	Highlights of Findings	Ref.
Ceftazidime	SD	Valine, leucine, methionine, phenylalanine, and tryptophan	Monophasic amorphous systems developed; nontoxic; leucine improved FPF; and tryptophan enhanced stability	[83]
Ceftazidime-roflumilast	SD	Leucine, tryptophan	Roflumilast increased solid-state dynamics but retained bioactivities; leucine significantly improved FPF	[84]
Ciprofloxacin	SD	Methionine, phenylalanine, and tryptophan	Improved antibiofilm activity and moisture protection due to D-amino acids	[90]
Ciprofloxacin–copper	Jet milling	Calcium carbonate	Significantly reduced CFU of *P. aeruginosa*	[91]
Ciprofloxacin–*N*-acetylcysteine-curcumin	SD	Leucine	Spherical particle (<6 μm) with wrinkled and depressed surface; excellent aerosolization; FPF (68.93% to 77.75%); and satisfactory antibacterial effects at low doses	[87]
Levofloxacin	SD	Leucine	Optimum leucine (21.79%); optimized pH 5.98; and produced 54.38% FPF	[88]
Levofloxacin	SD	Arginine	30% ethanol produced better powder; FPF above 50%; and emitted dose > 95%	[92]
Polymyxin B	SD, followed by airflow milling	Lactose, mannitol	Spray-dried particle size 3 µm; FPF, 53%, and milling increased FPF by 20%; and bioavailability peak 77.46% within 10 min	[86]

CFU, colony-forming unit; FPF, fine particle fraction; SD, spray drying.

*b.*  *Release modified microparticles*

Biodegradable polymers have been widely employed to develop microparticles capable of modulating drug release kinetics (Table 3). The use of chitosan demonstrated notable versatility in DPI development, including enhancing antibacterial and antibiofilm activity [85,93], improving swelling properties [94], and fast dissolution [95,96]. Chitosan released the drug immediately, whereas combining chitosan with PLGA improved the drug release profile [97,98]. Alginate–carrageenan microspheres were used to encapsulate ciprofloxacin. The freeze-dried product released 72.64% of the drug within 10 min [99].

Lin et al. used silk fibrin with mannitol to modify the release of ciprofloxacin for the treatment of non-cystic fibrosis bronchiectasis. They demonstrated improvements in drug loading efficiency and mucus penetration with the formulation [100]. These findings highlight the potential of polymeric materials for particle engineering in *P. aeruginosa* infections. Nonetheless, a comprehensive evaluation of polymer degradation and excretion is essential to ensure safety in the context of chronic lung infections.

**Table 3:** Studies of microparticles of antibiotics using release-modified polymeric materials for dry powder inhaler (DPI) targeting *P. aeruginosa* infection from 2015 to 2025.

Drug Name	Preparation Method	Characterization/Tests	Highlights of Findings	Ref.
Doripenem	The drug-loaded chitosan microparticles by SD Additives: lactose, trehalose, and leucine	PCM: XRD, SEM, and drug release; APCM: NGI; in vitro antibacterial activity test; and cell viability assay	EE approximately 80%, leucine had the most impact on powder aerosolization; chitosan microparticles	[85]
Gentamicin	Freeze-dried PLGA large-porous particles were dispersed in gentamicin sulfate, and it was lyophilized again.	PCM: SEM, flowability, DSC, and in vitro drug release; APCM: NGI; and stability test	Spherical and highly porous particles; drug released up to 30 min; and approximately 40% FPF	[101]
Ciprofloxacin	The drug and chitosan mix embedded silver that was subjected to a coating by silica using SD	PCM: TEM, particle size, DSC, and XRD; APCM: TSI; microbial activity (MIC, MBC); and biofilm erosion assay	Excellent aerodynamic deposition, 3–4 times more effective against the growth of the bacteria and their biofilm	[93]
Ciprofloxacin	The drug encapsulated into alginate–carrageenan microspheres and undertaken for FD	PCM: SEM, EE, drug loading, swelling index, and in vitro release; in vivo deposition study	Particles were spherical, smooth, and size < 2 µm; EE (52.86% to 76.29%); and maximum release amount (72.64%) for a formulation in 10 min	[99]
Ciprofloxacin	Drug microparticles are prepared by SD Additives: silk fibrin, mannitol	PCM: SEM, AFM, and DVS; antimicrobial activity; cytotoxicity; inflammatory cytokine measurement; lung function evaluation; and histology	FPF (45.04%); antimicrobial activity and lung function unaffected; and excellent biocompatibility and biosafety	[100]
Clarithromycin	SD powder formulation Additives: leucine, chitosan	PCM: yield and drug content, SEM, DSC, dissolution, and particle size distribution; APCM: NGI; in vitro deposition study; and antibacterial test	73.3% FPF; MMAD 1.8 μm; deposition 8.7 μg/cm^2^ in 24 h; no toxicity; and wide spectrum of antimicrobial property was not interfered	[96]
Levofloxacin	Drug–PLGA (DP) microsphere preparation by FD Additive: lauric acid	PCM: SEM, TEM, XRD, TGA, FTIR, and drug release; EE; APCM: MMAD determination; and cytotoxicity study	Particle size close to 5 µm exhibited good drug loading and controlled release; no cytotoxicity	[97]
Levofloxacin	Drug-loaded crosslinked chitosan microspheres formed by SD	PCM: SEM, XRD, TGA, FTIR, swelling property, drug loading and entrapment efficiency, water content, and particle size; APCM: NGI; in vitro release; and antibacterial assay	Particle size 5 µm; excellent aerosolization; antibacterial activity was similar to free drugs; and suitable swelling property	[94]
Levofloxacin	Drug–chitosan (DC) and drug–PLGA (DP) microspheres development and FD	Pharmacokinetic studies	DC released the drug immediately and 71% bioavailable in ELF; DP released the drug slowly and ELF concentration maintained up to 72 h	[98]
Levofloxacin	Corrugated surface microparticles with drug, chitosan, and an organic acid by SD Additive: leucine	PCM: EE, SEM; AFM, FIB, DSC, size distribution, XRD, and FTIR; APCM: ACI; dissolution study; PIV; pharmacokinetic study; and BALF	Formic acid’s powder had the most FPF (41.3% ± 3.9%), better aerosolization; reduced x-axial velocity and variable angle; fast dissolution; and high bioavailability in EFL	[95]

ACI, Anderson Cascade Impactor; AFM, atomic force microscopy; APCM, aerodynamic property characterization method; BALF, bronchoalveolar lavage fluid; DSC, differential scanning calorimetry; DVS, dynamic water vapour sorption; ED, emitted dose; EE, encapsulation efficacy; ELF, epithelial lining fluid; FD, freeze drying; FIB, focused ion beam; FPF, fine particle fraction; FTIR, Fourier transform infrared spectroscopy; MBC, minimum bactericidal concentration; MC, moisture content; MIC, minimum inhibitory concentration; MMAD, mass mean aerodynamic diameter; NGI, next generation impactor; PCM, particle characterization method; PIV, particle image velocimetry; SD, spray drying; SEM, scanning electron microscope; TEM, transmission electron microscope; TGA, thermogravimetric analysis; XRD, X-ray diffraction; and TSI, twin stage impinger.

#### 4.2.2. Nanoparticles

Nanoparticles typically range in size from 1 to 1000 nm; however, for better penetration through lung biological barriers and improved stability, sizes closer to 1–200 nm are often preferred [102]. These nanoparticles comprise a wide range of advanced delivery carriers, for instance, engineered nanomaterials, polymeric nanoparticles, carbon nanotubes, mesoporous nanoparticles, nanomaterials, micelles, dendrimers, liposomes, niosomes, and metallic nanoparticles [103]. These systems enable precise control over key pharmacokinetic and pharmacodynamic parameters, including solubility, metabolism, elimination, and toxicity.

Inhalable nanoparticles offer substantial potential to enhance the diffusion of drugs across biological barriers, including the biofilm and bacterial membrane [104]. Nevertheless, only a few studies have utilized freeze-dried nanoparticles as dry powders for inhalation. For instance, Bhattacharya et al. produced freeze-dried powder of nanosized aztreonam vesicles (144 nm), achieving drug entrapment up to 75% and improving cellular uptake [105]. However, the study did not report findings on the powder’s aerodynamics. Topal et al. prepared ciprofloxacin-loaded poly(ε-caprolactone) nanoparticles and converted them into dry powder by freeze drying [106]. Due to the high mannitol content (1:5 and 1:10 *w*/*w*), the formulation was classified as nanocomposite microparticles and exhibited a fine particle fraction of approximately 20%.

#### 4.2.3. Nano-in-Microparticles

Nano-in-microparticles are drug delivery formulations in which nanoparticles are embedded within microparticles. This mitigates the drawback of nanoparticles alone, which can fail to deposit in the respiratory tract because of their size; their diffusion, sedimentation, and impaction characteristics are such that a large fraction of the inhaled dose is exhaled [107]. Moreover, nanoparticles may cause uncontrolled aggregation that leads to inconsistent lung delivery over time [108].

##### Lipid-Based Nano-in-Microparticles

Both spray drying and spray freeze drying have been employed to produce these microparticles. Most of the formulation techniques used cytoprotectants (mannitol, trehalose, and sucrose) as additives (Table 4). Microparticles with a size below 5 µm are suitable for inhalation and deep lung deposition. At the same time, lipid vesicles facilitate drug diffusion across cell membranes and enhance penetration into the biofilm [109].

The selection and combination of lipids are critical for meeting functional criteria, including size, encapsulation efficacy (EE), durability, and drug release. Studies that used optimized lipid compositions for their formulations are summarized in Table 4. For example, Bashi et al. used Lipoid SPC (100%), dimethyl dioctadecyl ammonium bromide (DDAB), and D-α-Tocopheryl Polyethylene Glycol 1000 Succinate in a proportion of 160:40:1 and encapsulated azithromycin using thin-film hydration followed by spray drying. The liposomal preparation method achieved 75% EE. The particles were physicochemically stable and showed enhanced activity against the clinical isolates of *P. aeruginosa* grown in biofilm [110]. Another study used phospholipon 90 G and cholesterol at a molar ratio of 1:1 to encapsulate polymyxin B and produced very small (250–550 nm) microparticles by spray drying [111]. The liposomal clarithromycin achieved an EE of 80%, while maintaining drug stability [112]. A nanostructured lipid carrier encapsulated 98.75% of ciprofloxacin, improving the antibacterial effect with controlled release and physical stability [113]. However, the highest FPFs reported in each study were less than 50% [110,111,112,113]. Khatib et al. used magnesium stearate with leucine to improve the aerosolization of their lipid-based nano-in-microparticles, achieving an FPF of approximately 70%. That study developed liposomal ciprofloxacin nanocrystals that exhibited prolonged drug release up to 12 h using a sucrose and lipids ratio of 2:1 [114].

Synergistic antibiotic effects can sometimes be achieved by combining antibiotics in a formulation. Two developmental studies used ciprofloxacin and colistin co-loaded liposomal nano-in-micro-formulations. Yu et al. prepared co-encapsulated liposomes via thin-film evaporation and sonication, followed by spray freeze drying, achieving strong antibacterial activity against clinical *P. aeruginosa* isolates, surpassing ciprofloxacin or colistin alone [115]. Another study developed a muco-inert liposomal preparation that effectively penetrated respiratory mucus and accumulated within the bacterial biofilm. The formulation eliminated 99.99% of biofilm-embedded persistent bacteria and significantly reduced bacterial colonization (by 99.7%), inflammatory response, and pulmonary fibrosis in an in vivo model [116]. Nonetheless, none of the studies could confirm whether the ratio of ciprofloxacin and colistin they used was synergistic.

**Table 4:** Studies of lipid-based nano-in-micro antibiotic preparation for dry powder inhaler (DPI) targeting *P. aeruginosa* infection from 2015 to 2025.

Drug Name	Preparation Method	Characterization/Tests	Highlights of Findings	Ref.
Azithromycin	The drug was loaded into liposomes, and dry powder was prepared by SD Additives: trehalose, leucine	Liposomal characterization: EE and in vitro release; PCM: LD, SEM; APCM: NGI; MIC and MBC determination; biofilm studies; bacterial uptake; cellular uptake; and cytotoxicity studies	75% EE, excellent stability; improved bactericidal effect; antibiofilm activity > 75% of liposome uptake by bacterial cells in 1 h; and no cytotoxicity	[110]
Clarithromycin	Liposomal clarithromycin preparation by ultrasonic SFD Additives: mannitol, sucrose	PCM: SEM, moisture absorption, EE, uniformity study, and DSC; stability study	EE up to 80%; narrow size distribution; high drug emission, FPF up to 50%; and maintained stability at 60% RH and 25 °C for 3 months	[112]
Polymyxin B	Liposome was prepared by film hydration method and subjected to SD Additives: isoleucine, valine, and mannitol	PCM: drug content, SEM, FTIR, DSC, drug release assay, MIC, and MBC; APCM: NGI	Spherical, particle size 250–550 nm; FPF was 25–26%; 90% release within 2 h; and total *P aeruginosa* killing time of 12 h	[111]
Ciprofloxacin	Liposomal drug nanocrystals are subjected to SD powder preparation Additives: sucrose, magnesium stearate, and leucine	PCM: cryo-TEM, DLS, EE measurement, particle size and distribution, MC, XRD, DVS, and in vitro drug release; APCM: NGI	Spherical particle with low water content; FPF (66–70%), EE (71–79%); and sucrose and lipids ratio 2:1 exhibited prolonged drug release	[117]
Ciprofloxacin	Nanostructured lipid carriers–drug undertaken for SD Additive: chitosan	PCM: SEM; particle size, drug release; APCM: NGI; and antimicrobial assay	EE (98.75% ± 0.048%); loading capacity (13.34 ± 1.92%); 80% release in 10 min; excellent particle size and texture; and better antibacterial activity than free drug	[113]
Ciprofloxacin, Colistin	Liposome preparation followed DPI preparation by SFD Additives: leucine, trehalose, and sucrose	Liposome characterization; PCM: XRD; APCM: MSLI; cytotoxicity, time kill antibacterial assay; and MIC measurement	ED > 95% and FPF ~50%; EE around 50%; no effect on lung epithelial growth; and synergistic antibacterial effect	[115]
Ciprofloxacin, Colistin	Muco-inert Cipro-Col-Liposome preparation followed DPI powder preparation by SFD Additives: sucrose, mannitol, trehalose, and leucine	Liposome characterization: MIC, biofilm eradication concentration measurement, protective effect on blood cell, and biofilm leakage capacity; PCM: SEM, density; APCM: NGI; mucus penetration and biofilm accumulation; and antibiofilm effect analysis	FPF around 45%; ED nearly 100%; effectively penetrated the airway mucus and accumulated at the biofilm site; synergistically reduced the biofilm; 99.7% reduction in bacterial colonization; and mitigated inflammation and pulmonary fibrosis	[116]

APCM, aerodynamic property characterization method; DLS, dynamic light scattering; DSC, differential scanning calorimetry; DVS, dynamic water vapour sorption; ED, emitted dose; EE, encapsulation efficacy; FPF, fine particle fraction; FTIR, Fourier transform infrared spectroscopy; LD, laser diffraction; MBC, minimum bactericidal concentration; MC, moisture content; MIC, minimum inhibitory concentration; MSLI, Multi-Stage Liquid Impinger; NGI, next generation impactor; PCM, particle characterization method; SD, spray drying; SEM, scanning electron microscope; SFD, spray freeze drying; TEM, transmission electron microscope; and XRD, X-ray diffraction.

##### Polymer-Based Nano-in-Microparticles

Nanoparticles of a drug can be embedded in polymer matrices, followed by a drying process to develop nano-in-microsystems (Table 5). The primary objective of this nanoformulation is to modify drug release [118,119]. A nanocomplex of tobramycin with polyanion (Poly(2-hydroxyethyl aspartamide)–ethylenediamine–glucuronic acid conjugate) was subjected to spray-dried microparticle preparation. Embedding the nanoparticles in mannitol-based microparticles showed significantly enhanced antibiofilm activity compared to tobramycin and TOBI Podhaler; however, the study did not report any drug release data [120]. A study produced PLGA-PEG-colistin nano-in-microparticles that exhibited a burst effect in drug release within 10 min [121]. Another study followed a straightforward co-assembly reaction between ciprofloxacin (CIP) and poly(2-ethyl-2-oxazoline) (PEtOx) using tannic acid and developed a polymer-linked nano-in-micro-formulation. It showed 78% of drug release in 168 h, with a burst release of 50% in the first 12 h [122]. The extent of conjugation may be a key determinant in the polymer-based production of controlled-release nano-in-microparticles.

### 4.3. Future Directions in Formulations of Pulmonary Antibiotics

Overall, there has been significant progress in the development of inhalable antibiotic formulations for *P. aeruginosa* pulmonary infection in recent years; however, the transition from a powder formulation at the bench to clinical trials and the bedside remains constrained by common challenges. These include variability in aerosol performance and limited drug penetration through mucus and biofilms.

Furthermore, it is notable that many inhalable antibiotics that are formulated undergo little or no further development. A clear line of sight is required from drug formulation to in vitro and in vivo pre-clinical studies and then to clinical trials. This is challenging, as it requires expanding collaborations and increasing resources at each step, but it is essential if progress is to be made.

## 5. Development of Pulmonary Biologics

### 5.1. Antibacterial Proteins/Peptides

Antimicrobial peptides and proteins are components of the innate immune system of a wide range of organisms, including humans, animals, plants, fungi, bacteria, and insects [123]. They exhibit inhibitory effects against a broad class of organisms, including viruses, bacteria, fungi, and helminths [124]. We and others are repurposing antimicrobial peptides to tackle antibiotic resistance [12,13,125,126,127] and to potentiate the action of antibiotics when co-administered [128,129,130].

A few studies on DPI formulations are available as a monotherapy or in combination with antibiotics, and several techniques have been used to develop the free-flowing, release-controlled delivery of the proteins, including polymeric carrier conjugation, liposomal incorporation, and nanosized particle optimization (Table 6).

Innate Defence Regulator-1018 (IDR-1018) and *N*-acetyl cysteine (an antituberculosis drug) were embedded into porous PLGA microspheres and subjected to spray drying. The developed powder exhibited FPF above 50% and the potential to cross a mucosal barrier, demonstrating efficient bactericidal activity, biofilm disruption, and an anti-inflammatory response [131]. Another slow-release system was developed using PLGA to minimize the toxicity of peptide SET-M33. The study developed nanosized particles (200 nm) with dose-dependent bactericidal effects on *P. aeruginosa* up to 72 h but no toxicity to bronchial epithelial cells, unlike the free peptide [132]. Lysozyme-loaded polycaprolactone (PCL) was used as a carrier to formulate lysozyme microparticles via the solvent evaporation method, followed by lyophilization. A sustained release system showed protein release up to 35 days, with an initial burst effect [133].

The liposomal incorporation of drugs reduces protein toxicity through a slow-release profile and facilitates drug diffusion across biological barriers. Serratiopeptidase and levofloxacin were loaded into liposomal carriers. Their developed system eradicated 90% of the biofilm at sub-MIC levofloxacin concentrations and decreased the mRNA expression associated with inflammation [134]. Casciaro et al. developed nanoparticles of Esculentin-1a derivatives [Esc(1-21) and Esc(1-21)-1c] using carrier PLGA [135]. Particle sizes were between 261 nm and 282 nm and easily diffusible within the mucus layer. Intratracheal administration showed a 3-log reduction in the bacterial load of the lung within 36 h for a single dose.

Collectively, DPI peptide formulations have utilized polymeric carriers, liposomal systems, and nanoparticle developments. PLGA microspheres and polycaprolactone carriers demonstrated sustained release, biofilm disruption, and reduced toxicity, while liposomal systems enhanced anti-inflammatory effects and synergistic antibiotic action. Moreover, promising in vivo outcomes included significant bacterial load reduction and improved lung compatibility, highlighting their potential for pulmonary delivery to treat *P. aeruginosa* infections.

### 5.2. Vaccines and Antibodies

Vaccines and antibodies are mainly delivered by parenteral routes. Some reports on the mucosal delivery of vaccines have demonstrated the efficacy of eliciting a prompt immunological response to eradicate bacterial loads in the lungs. Pneumococcal subunit polysaccharide and protein vaccines are mainly used to prevent pneumococcal pneumonia. Nakatsuka et al. demonstrated the effectiveness of lung *P. aeruginosa* clearance when this vaccine was delivered via the intratracheal route. They administered heat-killed *P. aeruginosa* and proved the prolonged disappearance of Regnase-1, an endoribonuclease expressed in airway epithelial cells and immune cells, whereas only transcription factor NF-κB was transiently active. The epithelial cell-specific deletion of Regnase-1 augmented both innate defences against *P. aeruginosa* and enhanced the secretion of the bacteria-specific IgA and Th17 accumulation in the lung [136].

*Escherichia coli* was bioengineered to assemble biopolymers that were coated with epitopes (Ag) derived from proteins primarily associated with *P. aeruginosa* outer membrane proteins, including OprF, OprI, AlgE, OprL, PopB, PilA, PilO, FliC, and Hcp1. Application of the vaccine in mouse models showed that particulate vaccines induced cellular and humoral responses, resulting in up to 90% survival. Moreover, the study proved that the intranasal route exhibited better prevention of *P. aeruginosa* infection than intramuscular delivery [137].

Antibodies neutralize pathogens and enhance the recruitment of effectors to the site of the pulmonary infection, facilitating bacterial elimination. Monoclonal antibody mAb166 was applied after primary and secondary infection by three strains of *P. aeruginosa* (PA103, CLJ1, and PA14). Initially, PA103 was applied at LD_100_, and after 1 h, the antibody was applied. At 30 days, the surviving mice were rechallenged for secondary infection using all three strains. The group treated with monoclonal antibodies had a significant survival rate in secondary infection without antibody delivery. In vitro antigen-presenting cells (APCs) stimulation assay, in vivo *P. aeruginosa* challenges, and serum transfer tests revealed a crucial involvement in antibody and bacterial interactions associated with a sustained humoral response [138].

The efficacy of the airway administration of mAb166 has been shown to be better than parenteral routes in a murine model of lung infection with the *P. aeruginosa* strain PA103. Antibody was applied two hours before the *P. aeruginosa* infection, and the bacterial loads, cytokines, chemokines, and other proteins were measured. The antibody ratio between airways and blood was 10-fold for airway administration, which resulted in a higher survival time, with about 80% survival up to seven days of infection. In contrast, other routes showed a rate below 60%. Additionally, fewer inflammatory mediators and neutrophil infiltration were detected in the bronchoalveolar lavage during respiratory delivery [139].

In summary, respiratory mucosal delivery of vaccines has revealed the superior immune activation and lung clearance of bacteria, as demonstrated by the increased secretion of IgA and Th17 and survival rate of animals compared to parenteral routes. In addition, utilizing the respiratory route for mAb delivery provided strong protection against *P. aeruginosa*, with higher local concentrations and substantially increased survival.

### 5.3. Bacteriophages

Bacteriophages are viruses that are considered to be promising agents for killing resistant bacteria when antibiotics fail. A recent study by Wannigama et al. showed that intranasal delivery enhanced pulmonary localization of the phage, minimized antibody-mediated neutralization, and enhanced therapeutic efficacy against *P. aeruginosa* lung infections [140]. Case reports have indicated the safety and efficacy of phage delivery to the lung to treat MDR *P. aeruginosa* [141,142,143].

This work mainly utilized spray drying techniques for DPI powder formulations with excellent aerosolization properties. Given phage viability concerns, those investigations focused on the optimization of storage conditions and thermal stability during spray drying. As a result, dry powders of the *Pseudomonas* phage (PEV2) were prepared in the matrices of leucine, trehalose, and mannitol and evaluated for stability. Humidity had a destructive effect on phage viability, but trehalose contributed significantly at 0 and 22% RH at 4 °C for 12 months [144]. A recent study showed that high concentrations of lactose significantly impacted the long-term stability (4 years) of phage powder (PEV1, PEV20, and PEV61) at a relative humidity of <15% and 4 °C [145]. Another study showed that hydrolyzed gelatine was the most effective bacteriophage stabilizer during spray drying, outperforming trehalose, lactose, leucine, mannitol, and gelatine. In addition, both hydrolysed and non-hydrolysed gelatine showed the highest stability at 4 °C [146]. Li et al. combined human serum albumin and lactose to improve phage stability and achieved a reduction in phage viability below 0.8 log_10_, with a combination of 60% and 40%, respectively [147]. To escape the stresses of drying, liposomal phage nanoparticles were developed that reduced cellular uptake (2-fold) and extended extracellular retention of the bacteriophage in lung epithelial cells [148].

Chang et al. applied a *Pseudomonas* phage spray-dried powder in a murine infection model to evaluate the bactericidal effects against MDR *P. aeruginosa*. Phage PEV20 was used to prepare powders using the matrices of lactose and leucine, and the powders were applied intratracheally 2 h after challenge with the *P. aeruginosa*-resistant strain FADDI-PA001. Twenty-four hours later, a significant reduction in the infected bacteria by 5.3 log_10_ was measured, compared with the control group. Additionally, the bacteriophage count was increased by 1 log_10_ [149]. Another study showed a >4 log reduction in bacterial growth against the multidrug-resistant strain FADD1-PA001 by combining anti-pseudomonas phage PEV31 with ciprofloxacin. Moreover, the sample was aerosolized using a vibrating-mesh nebulizer, and the collected samples demonstrated efficacy in inhibiting bacterial density [150].

Inhaled formulations of bacteriophages show promise for the treatment of MDR *P. aeruginosa* infections; in vivo models have shown a significant reduction in bacterial loads and synergistic effects with antibiotics. Further work on formulation stability is needed to optimize phage viability during storage. Bacteriophage stabilizers and liposomal encapsulation may mitigate the need to control the storage temperature and humidity within narrow limits, which would limit clinical utility.

### 5.4. Probiotics

Healthy respiratory mucosa maintains a balanced and varied microbiota, acting as a defence against pathogens. The composition of the lung microbiota established during birth is influenced by several factors, including diet, environmental exposures, and antibiotics [151]. The microbiota of the GIT influences the respiratory mucosal environment because of the direct exchange of microbes and their products through lymphatic circulation [152]. The dysbiosis of the lung microenvironment in chronic lung disease elicits an inflammatory response and facilitates infection, and oral probiotics can limit this dysbiosis and regulate innate immune responses. Common microbial genera (e.g., *Lactobacillus*, *Bifidobacterium*, and *Saccharomyces*) are used as GIT supplements and have been found to influence the lung microbiota.

Studies have shown the protective role of probiotics against *P. aeruginosa*. Probiotics containing *Bifidobacterium longum* and *Lactobacillus rhamnosus* modulate beta-defensin-2 and inflammatory IL-8 during *P. aeruginosa* infection [153]. Oral probiotics also affect respiratory resistance to *P. aeruginosa* colonization: a meta-analysis of patients in the intensive care unit of a hospital showed that the time to infection was significantly delayed in patients given the oral probiotic *Lactobacillus rhamnosus* [154]. A small number of studies have examined probiotic delivery to the respiratory tract. For example, intranasal delivery of a *Lactobacillus* spp. resulted in significant clearance of *S. pneumoniae* from the lung in a murine model: Swiss albino mice treated with intranasal *Lactobacillus* spp. showed decreasing bacterial CFUs and reduced lung injury compared with the control group when challenged with *S. pneumoniae* [155].

The formulation of probiotic dry powder for combating *P. aeruginosa* infection was further explored by Glieca et al. and Tran et al. Spray-dried inhalable dry powders of *L. plantarum*, *L. rhamnosus*, and *L. acidophilus* were prepared, preserving bacterial viability. Three concentrations of CFU 10^9^, 5 × 10^9^, and 10^10^ CFU/mL were dispersed into PBS or Ringer’s solution and taken for spray drying at a 135 °C inlet temperature. Lactose and leucine at a 70/30 ratio were used as a matrix for bacterial colony. The bacteria withstood the processing parameters, decreasing by only 1 log CFU, and that was maintained for up to a month. Particle characterization confirmed the respirability of powder and its potential for lung deposition. *L. plantarum* powder exhibited a potential antibacterial effect against *P. aeruginosa*. Interestingly, the lactic acid bacteria survived at an inlet temperature of 135 °C, although no report was found that these bacteria are viable up to 100 °C [156]. The excipients probably made a protective layer on the colony of the bacteria. To avoid the thermal detrimental effects on probiotics, the SFD method was adopted to prepare the DPI of *Lactobacillus rhamnosus GG* (LGG). However, the optimized formulation (LGG–leucine–lactose–mannitol = 35: 5: 15: 45 *w*/*w*%) exhibited a 0.9 log reduction in cell viability [157]. Apart from heat, other processing parameters may influence cell viability and should be systematically evaluated in future studies.

Oral probiotics can be employed to modulate immune responses, reduce bacterial load, and improve resistance to *P. aeruginosa* infections in the lungs. The relationship between the GIT and lung microbiomes, however, remains only partially understood, and it is unclear whether the translocation of healthy gut bacteria or their products from the GIT to the lungs is more important. As a greater understanding of the gut–lung axis emerges, so will opportunities for inhaled probiotics and their products.

## 6. Prospects for Synergistic Combinations of Antibiotics and Biologics

Synergistic combination is a delivery strategy to improve the bactericidal effects of two or more agents by more than the sum of their effects by the mutual or one-sided modulation of pharmacokinetics and pharmacodynamics. Proteins and other biologics commonly interfere with antibiotic uptake, immune response, and bacterial enzymatic protection, potentiating activity.

Two brevinin-1 peptides were combined with kanamycin, ciprofloxacin, and tetracycline and exhibited potentiation of drug activity against *P. aeruginosa* by decreasing the MIC 4–25-fold compared to the drug alone [158]. Similarly, another study reported the potential synergistic effects of the IB-367 protein with imipenem and colistin against 24 g-negative strains of bacteria, including six of *P. aeruginosa* [159]. In a bacteriophage synergy evaluation, the phage of *P. aeruginosa* (EPA1) was applied on a 48 hr old biofilm with seven antibiotics, such as ciprofloxacin, meropenem, tetracycline, erythromycin, gentamicin, kanamycin, and chloramphenicol, and showed a synergistic reduction in bacterial counts on biofilm [160]. Lin et al. used a powder of the synergistic combination of bacteriophage (PEV20) and ciprofloxacin in an acute lung infection model for neutropenic mice. The combination significantly decreased the bacterial loads of *P. aeruginosa* in the lungs by 5.9 log_10_ (*p* < 0.005), whereas PEV20 or ciprofloxacin alone did not cause an obvious reduction [161]. In addition, probiotics (*Lactobacillus casei*, *L. rahmnosus*) with aminoglycosides synergistically reduced the MIC and MBC of *P. aeruginosa* [162].

Innate Defence Regulator-1018 (IDR1018) was investigated for synergistic anti-TB activity combined with *N*-acetyl cysteine (NAC). The PLGA microsphere formulation exhibited significantly increased mucosal barrier disruption by 4.1-fold and reduced the bacterial load by approximately 3.02 log CFU/mL [131]. Thus, the synergistic combination may potentiate antibiofilm activity, which is important for *P. aeruginosa* eradication from the respiratory tract.

Research should influence antibiotic–peptide synergy by defining critical synergy thresholds as the synergy-defining Fractional Inhibitory Concentration Index (FICI), optimizing pharmacokinetic/pharmacodynamic targets, integrating regulatory control frameworks, and addressing priority gaps in resistance management to guide pulmonary product development and clinical application. It is not just a way to enhance drug activity but a strategy to minimize the need for novel antibiotic molecules to manage the drug resistance crisis.

## 7. Conclusions and Future Perspectives

Treating *P. aeruginosa* infections is challenging due to its intrinsic structural and functional resistance mechanisms. The delivery of antibiotics and biologics via pulmonary routes offers a promising strategy to overcome the limitations of conventional methods, including maintaining high local drug concentrations, improving penetration into biofilms, and achieving sustained drug release. The reviewed evidence highlights significant advances in particle engineering for dry powder inhalers, nebulized formulations, and advanced nano-in-microsystems, which enhance drug deposition and biofilm penetration. Biologics, including antimicrobial peptides and bacteriophages, offer innovative ways to combat multidrug resistance.

There are some research gaps that require focused attention to use the inhaled route effectively. Only a limited number of anti-pseudomonal drugs have been successfully translated into pulmonary products, indicating opportunities for further investigation. The synergy between antibiotics and biologics shows promise for enhancing antibacterial and antibiofilm activities against MDR *P. aeruginosa*. In addition, ensuring effective and safe pulmonary delivery requires a rational approach that prioritizes stringent regulatory compliance for both drugs and additives, considering the sensitivity of lung tissue regarding allergenicity, irritancy, and toxicity. Current evidence indicates a gap in efficacy studies against clinical isolates, which are crucial for understanding dose adjustments across different resistance levels. Overcoming these limitations through careful pulmonary product development and aligning clinical research will be essential for translating promising formulations into effective, safe, and reliable therapies.

## Figures and Tables

**Table 5:** Studies of polymer-conjugated nano-in-micro antibiotic dry powder inhaler (DPI) formulations targeting *P. aeruginosa* infection from 2015 to 2025.

Drug Name	Preparation Method	Characterization/Tests	Highlights of Findings	Ref.
Tobramycin	Drug and polyanion (PHEA-EDA-GlucA) nano complex subjected to microparticle formulation by SD Additives: mannitol *N*-acetylcysteine, and arginine	PCM: SEM, drug content, mucus penetration assay, cell viability, MIC, MBC, and biofilm inhibition assay	Spherical, size approximately 2.5 µm, high cytocompatibility to lung epithelial cells, and pronounced antimicrobial and antibiofilm activity	[120]
Colistin	PLGA-PEG-col nanoparticles subjected to microparticle preparation by electrospray	PCM: TEM; APCM: NGI; and antibacterial assay	Antisolvent precipitation exhibited the highest drug loading capacity (55%); excellent MMAD; and complete bacteria eradication	[121]
Ciprofloxacin	Drug-loaded poly (2-ethyl-2-oxazoline) nanoparticles subjected to SD powder preparation	PCM: particle size, density, flow property, loading dose, DSC, TGA, and release FTIR; APCM: TSI	21–67% of drug loading; maximum 78% released in 168 h with a burst release of 50% in the first 12 h; and FPF (34.4% and 40.8%)	[122]

APCM, aerodynamic property characterization method; DSC, differential scanning calorimetry; FPF, fine particle fraction; FTIR, Fourier transform infrared spectroscopy; MBC, minimum bactericidal concentration; MIC, minimum inhibitory concentration; MMAD, mass mean aerodynamic diameter; NGI, next generation impactor; PCM, particle characterization method; SD, spray drying; SEM, scanning electron microscope; TEM, transmission electron microscope; TGA, thermogravimetric analysis; and TSI, twin stage impinger.

**Table 6:** The development of pulmonary products of antimicrobial proteins/peptides targeting *P. aeruginosa* infection and biofilm.

Name	Objective	Research Design	Findings	Limitations	Ref.
Innate Defence Regulator-1018 (IDR1018)	Evaluating anti-TB activity of N-acetyl cysteine (NAC) with IDR1018 embedded into porous PLGA microspheres. Endeavour to find the synergistic effects with the mucus-penetrating and biofilm-disrupting properties.	Incorporation of the protein into porous PLGA microspheres and subjected to NAC-coated particle preparation and freeze drying; PCM: size distribution, shape, surface area, porosity, EE, and release profile; APCM: NGI; particle permeation analysis; in vitro phagocytosis, antibiofilm, and mycobactericidal cell viability assay. In vivo inhaled dose, particle retention estimation; cytokine level, histopathology, and bacterial load in the lungs and spleen.	MMAD 3.79 ± 1.04 μm; FPF 52.9 ± 5.11%; particles transit through mucosal barrier was increased by 4.1-fold; disrupted the bacterial biofilm; reduced bacterial load by approximately 3.02 log CFU/mL; reduced the inflammatory response in the lung; and significantly decreased necrotizing lesions in dose–response manner.	The study does not exactly represent the *P. aeruginosa* biofilm-disrupting capacity.	[131]
Peptide SET-M33	Developing a slow-release drug delivery to the lung to reduce the toxicity of peptide SET-M33.	The protein-loaded PEGylated PLGA nanoparticles (4 mg of SET-M33 per 100 mg of NPs); PCM: PDI, DLS, ELS, TEM, EE, and release study; APCM: NGI; in vitro interaction with mucin and alginate, transport to mucus and biofilm; antibacterial activity against PA; and antifilm, cytotoxicity, and in vivo toxicity studies.	Particle size was around 200 nm; prolonged antibacterial properties; lower toxicity; dose–response bactericidal effects on *P. aeruginosa* to 72 h, no cytotoxicity on bronchial epithelial cells, and unlike the free peptide; and no appreciable side effects.	No study on in vivo antibiofilm assay.	[132]
Lysozyme	Optimization of the lysozyme delivery in the lung against cystic fibrosis.	Lysozyme-loaded polycaprolactone (PCL) microparticles were prepared using solvent evaporation method and lyophilization; PCM: particle size, EE, zeta potential, FTIR, SEM, in vitro release, SDS-PAGE analysis, and stability study; and APCM: ACI	Spherical and smooth particles, size 8.75 µm, and EE 65.15%; sustained release up to 35 days with initial burst release; MMAD 5.44 ± 0.19 μm; and FPF 50.99 ± 2.89%.	No in vitro or in vivo studies on antibacterial, antibiofilm, and toxicity studies.	[133]
Serratiopeptidase	Analyzing the effect of the protein with levofloxacin liposomes on biofilm eradication.	Levofloxacin liposome preparation and SD powder preparation with the protein; PCM: SEM, XRD, MC, and PCS; APCM: ACI; pharmacodynamic studies; microbial burden and immunological markers calculation, pharmacokinetic studies; and histopathological assay on rat infection model of *S. aureus.*	The combination eradicated 90% biofilm at sub-MIC of levofloxacin. EE of LFX 80%; MMAD < 5 μm. Lower mRNA expression responsible for inflammation; mild hyperplasia in lymphoid tissue of bronchi.	The study does not exactly represent the *P. aeruginosa* biofilm-disrupting capacity.	[134]
Esculentin-1a Derivatives [Esc(1-21) and Esc(1-21)-1c (Esc peptides)]	Evaluation of the bactericidal effects of Esc peptides loaded PLGA nanoparticles.	Nanoparticles production and FD; PCM: PDI, DLS, ELS, TEM, zeta potential, in vitro release kinetics, and interaction with mucin and alginates transport through mucus and biofilm; APCM: NGI; and antimicrobial activity against *P. aeruginosa*; in vivo efficacy and toxicity assay.	Sizes were 261 nm and 282 nm, respectively; zeta potentials were suitable for dispersibility; easily diffusible to mucus layer; and lung bacterial load was reduced by 3 log within 36 h after a single dose administration intratracheally.	No information on the lung retention and clearance times.	[135]

ACI, Anderson Cascade Impactor; APCM, aerodynamic property characterization method; DLS, dynamic light scattering; EE, encapsulation efficacy; ELS, electrophoretic light scattering; FD, freeze drying; FPF, fine particle fraction; FTIR, Fourier transform infrared spectroscopy; MC, moisture content; MIC, minimum inhibitory concentration; MMAD, mass mean aerodynamic diameter; NGI, next-generation impactor; PCM, particle characterization method; PCS, photon correlation spectroscopy; PDI, polydispersibility index; SD, spray drying; SDS-PAGE, sodium dodecyl sulphate-polyacrylamide gel electrophoresis; SEM, scanning electron microscope; TEM, transmission electron microscope; and XRD, X-ray diffraction.

## Data Availability

No new data were created or analyzed in this study.
